# The Stanford Data Miner: a novel approach for integrating and exploring heterogeneous immunological data

**DOI:** 10.1186/1479-5876-10-62

**Published:** 2012-03-28

**Authors:** Janet C Siebert, Wes Munsil, Yael Rosenberg-Hasson, Mark M Davis, Holden T Maecker

**Affiliations:** 1 CytoAnalytics, Denver, CO, USA; 2 The Institute for Immunity, Transplantation, and Infection, Stanford University, Stanford, CA, USA; 3 The Department of Microbiology and Immunology, Stanford University, Stanford, CA, USA; 4 The Howard Hughes Medical Institute, Chevy Chase, MD, USA

**Keywords:** Systems immunology, Data integration, Data warehousing, OLAP

## Abstract

**Background:**

Systems-level approaches are increasingly common in both murine and human translational studies. These approaches employ multiple high information content assays. As a result, there is a need for tools to integrate heterogeneous types of laboratory and clinical/demographic data, and to allow the exploration of that data by aggregating and/or segregating results based on particular variables (e.g., mean cytokine levels by age and gender).

**Methods:**

Here we describe the application of standard data warehousing tools to create a novel environment for user-driven upload, integration, and exploration of heterogeneous data. The system presented here currently supports flow cytometry and immunoassays performed in the Stanford Human Immune Monitoring Center, but could be applied more generally.

**Results:**

Users upload assay results contained in platform-specific spreadsheets of a defined format, and clinical and demographic data in spreadsheets of flexible format. Users then map sample IDs to connect the assay results with the metadata. An OLAP (on-line analytical processing) data exploration interface allows filtering and display of various dimensions (e.g., Luminex analytes in rows, treatment group in columns, filtered on a particular study). Statistics such as mean, median, and N can be displayed. The views can be expanded or contracted to aggregate or segregate data at various levels. Individual-level data is accessible with a single click. The result is a user-driven system that permits data integration and exploration in a variety of settings. We show how the system can be used to find gender-specific differences in serum cytokine levels, and compare them across experiments and assay types.

**Conclusions:**

We have used the tools and techniques of data warehousing, including open-source business intelligence software, to support investigator-driven data integration and mining of diverse immunological data.

## Background

Increasingly, translational studies in medicine take a "systems biology" approach, meaning that comprehensive measurements of many parameters are made, not just those hypothesized to be directly involved in the condition being studied. To accomplish this, multiple analytical platforms are often employed, such as gene expression, flow cytometry, and immunoassays. The data from all these platforms must then be integrated, along with metadata (such as clinical and demographic information), to understand the results as a whole.

As an example of a systems-level immunology study, we are currently investigating the effects of aging on immune responsiveness, as measured by influenza vaccination. In this study, healthy participants across a range of ages have blood samples taken at three time points before and after being administered an influenza vaccine. Responsiveness is measured by hemagglutinin inhibition (HAI). The particpants' immune systems are characterized at the cellular level by flow cytometry, at the genomic level by gene expression microarray, and at the soluble protein level by Luminex assays for serum cytokines. Relevant clinical parameters include age, gender, ethnicity, and the type of vaccine administered. All these variables need to be analyzed in a combined fashion. Similar studies are underway at other centers [1], and these data could potentially be integrated for additional mining.

Tools to easily integrate disparate data types and mine their combined results are currently not well developed for biology or medicine. We therefore set out to create a system for data integration and mining that would allow for the comparison of data across assay platforms and even across studies. Our requirements for the system included: (1) a reasonable development time and cost; (2) user-driven data management, with minimal programmer intervention; (3) support for user-driven data exploration with a shallow learning curve; and (4) support for multiple investigators and projects. We refer to the resulting system as the SDM for "Stanford Data Miner".

To accomplish these goals, we leveraged established techniques of data warehousing, a discipline designed to integrate heterogeneous data in a consistent fashion in support of analysis and decision making [2,3]. A data warehouse has three main components: the dimensional model, back-end processes for data integration, and end-user tools for exploration and analysis. We discuss each of these components in the following sections. In addition, we used open source software for the database, the web application server, and interactive data analysis. Furthermore, we deployed the system on cloud servers for ease of access, speed of procurement, and low ongoing costs.

## Methods and materials

The work is implemented as two web applications ("web apps") running under JBoss AS, an open source Java-EE-based application server, on a Rackspace Cloud Server running Ubuntu Linux. One web app is for data integration; the other is for data exploration. Source code is available at http://stanfordminer.sourceforge.net.

### Data integration web app

This is a fully custom Java "Web 2.0" product called Sherpa. Sherpa consists of around 11,000 lines of Java, 2,000 lines of XHTML (facelets), and 2,000 lines of other text (mostly properties and configuration files). Though fully custom, it is built using several open-source frameworks and toolkits. Chief among these is Seam, a platform integrating Asynchronous JavaScript and XML (AJAX), JavaServer Faces (JSF), the Java Persistence API (JPA), and Enterprise Java Beans (EJB) 3.0. From the JBoss RichFaces project (an advanced user interface component framework), Sherpa draws user interface components such as the calendar, the tab panel, the modal panel, and the extended data table, as well as AJAX support through the project's a4j library. Sherpa's drag-and-drop capability is provided by the open source JavaScript toolkit Dojo, extended for Sherpa's specific needs. Sherpa accesses Excel spreadsheets by means of Apache POI, a Java API for Microsoft documents.

### Data exploration web app

This is an open source business intelligence product called JasperServer (version 3.7), customized through supported configuration changes. JasperServer incorporates the Mondrian OLAP implementation, with a JPivot user interface. The OLAP interface in JasperServer provides functionality through a logical layer on top of an underlying relational database, in contrast to OLAP implementations requiring a special purpose data storage format. Thus it is known as relational OLAP, or ROLAP. Mondrian schemas, defined in XML, map the underlying database structure into an OLAP structure. Both web apps use the open source database engine MySQL for back-end data persistence.

Users authenticate to the two web apps individually, by user name and password. All web access to the Cloud Server uses HTTPS. Command-line access is through SSH, with only passwordless (public key) authentication allowed.

### Luminex assays

Data shown in the Results is based on a study of serum from 434 healthy participants, each sampled three times (before and 7 and 28 days post-flu vaccination). Luminex assays were performed using a custom 51-plex kit from Affymetrix (Santa Clara, CA). A list of analytes and protocol is available at http://iti.stanford.edu/research/himc-protocols-immunoassays.html. Some samples were tested twice using a second batch (different lot) of Luminex kits. Values from all three visits were included in the data shown.

### MesoScale Discovery (MSD) assays

These were performed according to the manufacturer's instructions http://www.mesoscale.com. A 4-plex cytokine assay (IL-1β, IL-6, IL-8, TNF) was performed on the same healthy participants at the same timepoints as described for Luminex assays above.

### Phosphoepitope flow cytometry assays

These were performed as described http://iti.stanford.edu/research/himc-protocols-flowcytometry.html. Briefly, cryopreserved PBMC were thawed, rested 1 h at 37°C, and stimulated for 15 min using saturating amounts of individual cytokines (IFNα, IFNγ, IL-2, IL-6, IL-7, IL-10, or IL-21) or left unstimulated. They were then fixed, permeabilized, and stained for surface markers and phospho-STAT1, phospho-STAT3, and phospho-STAT5.

All human studies were conducted under protocols approved by Stanford's Institutional Review Board, with written informed consent obtained from all participants.

## Results

### Data model

**Figure 1:**
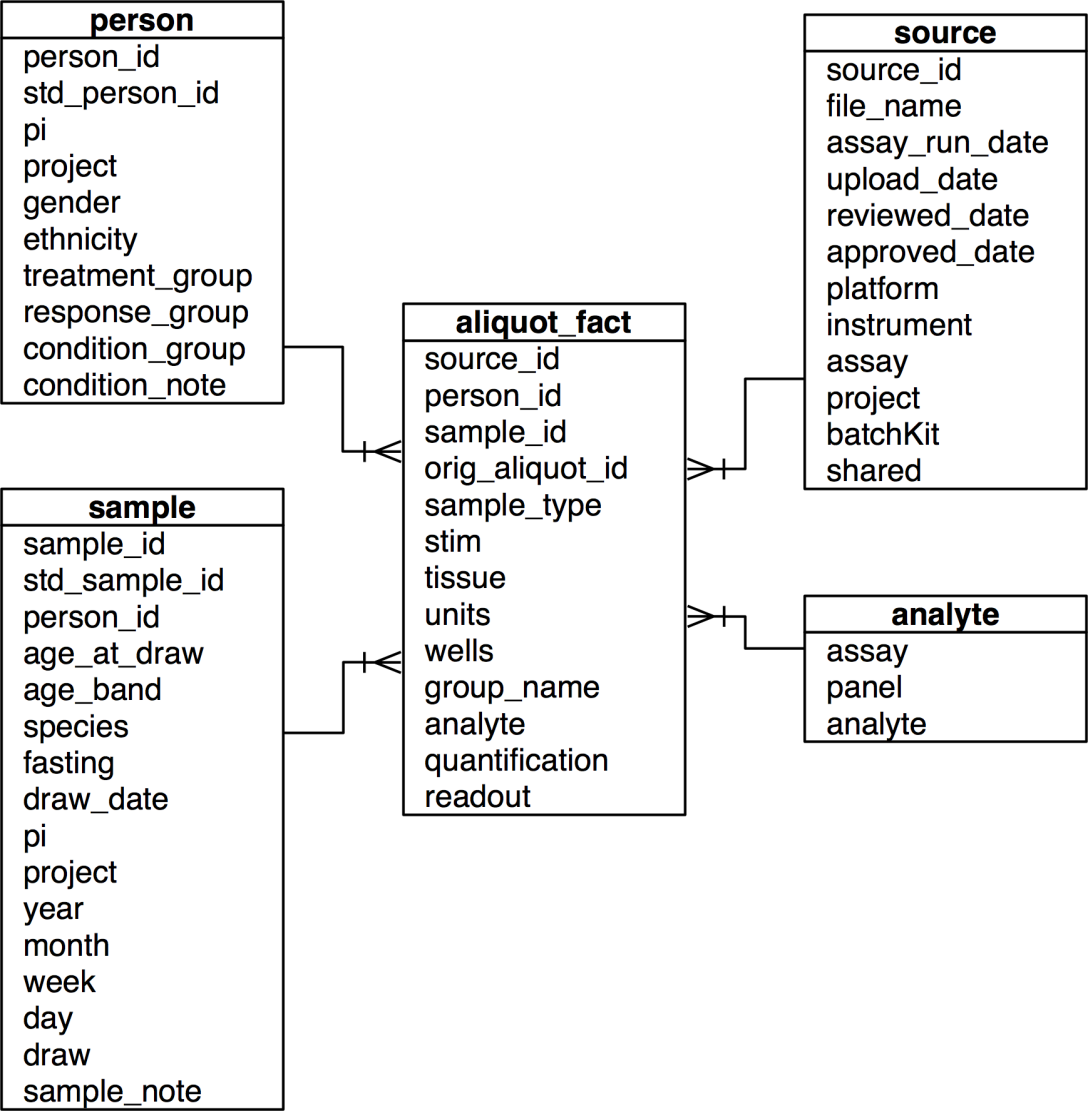
**Dimensional data model**. An aliquot_fact, or single experimental data point, is associated in our dimensional model with the additional dimensions of person, sample, analyte, and data source. This schema provides a simple way to represent and associate heterogeneous translational data.

One important goal of this work was to facilitate data analysis. Thus we employed dimensional modeling, a well-established technique from data warehousing. Dimensional modeling has been in use since the late 1960s [2]. The technique organizes data in a way that is easy to understand and provides high-performance access. In a dimensional model, a numeric fact about the domain is described by many attributes, called dimensions. As illustrated in Figure 1, our dimensional model is centered on the aliquot_fact table, which is further described by parameters describing the person, sample, analyte, and source document. Dimensional models are sometimes called star schemas because the illustration of the model resembles a star.

The aliquot_fact table, in conjunction with the analyte table, is designed to hold numeric results from multiple assays. The three main fields that support this are analyte, readout, and units. This generic structure supports results from diverse assays such as Luminex, flow phenotyping, and phospho-flow; and is easily extensible to more assays. Luminex data includes analytes such as CD40Ligand, IL-6, and VEGF; and units such as raw MFI, average MFI, and pg/ml. Flow phenotyping data includes analytes such as Lymph/CD3+/CD4+ and Lymph/CD3+/CD8+/CD28+ and units such as percent of parent population. This table also has a field for quantification (in range, below LOQ (limit of quantification), above LOQ), which we use to classify results from the Luminex and MSD calibration algorithms.

Tissue and stim, two essential features of these aliquots, are included in the aliquot_fact table. Tissue includes such values as cryopreserved PBMC, cryopreserved serum, and fresh plasma. Tissue is recorded at an aliquot level since a single sample (e.g. whole blood) can be processed into aliquots of different tissues such as PBMC and serum. Stim includes values such as IFNγ, IL-2, and IL-10, thereby capturing different stimulation conditions used in functional assays. The aliquot_fact table joins to the analyte, person, sample, and source tables with foreign keys.

The sample table includes the time point hierarchy, consisting of the fields year, month, week, day, and draw. These fields do not record calendar dates. Instead, they record the sequence and structure of study events. For example, in some studies, samples might be drawn on day 0, day 1, day 7, and day 28. These values are recorded in the day field. In a study that followed the same patient from year to year, the value for year would be recorded in the year field. In another study, samples might be drawn at week 0, week 5, and week 12. These values are recorded in the week field. In a third study, samples might be drawn on 3 subsequent days (day 1, day 2, and day 3), 3 different times a day (e.g., 10:00 AM, 12:00 PM, and 2:00 PM). Thus, our time point hierarchy supports many different study designs while capturing the natural structure of time.

The person table is designed to capture time-invariant characteristics of study participants that might be relevant to interpretation of study results. These include gender, ethnicity, and condition. While condition is often equivalent to disease, the field is designed to also support more general classifications (e.g. pregnant, antibody-positive). The person table also includes attributes for treatment and response. Treatment values could be as general as treated and placebo, or more study-specific such as cohort A, cohort B, and cohort C. Similarly, response could be as generic as responder and non-responder, or more specific such as partial response, complete response, stable, or progressive disease.

The source table records information about the batch results spreadsheets that contained the original data loaded into the database. Important attributes include file name, upload date, assay (e.g. Human Luminex 51plex, Flow phenotyping, Cytokine-stimulated phospho-flow), and assay run date. These fields support minimal information standards such as MIATA [4] and MIFlowCyt [5]. Additionally, the source table includes PI (principal investigator) and project, allowing us to group data based on a specific project or study, and on PI.

### User-driven data integration

The second component of a data warehouse is the set of backend processes for transforming and loading data into the dimensional model. Two related goals of this work were to accomplish data integration within a reasonable time and cost, and to support user-driven integration with minimal programmer intervention. This section describes the web-based user interfaces that allow users to upload batch results from sets of aliquots analyzed with a particular assay, and to upload metadata describing persons and samples. In both cases, the data is uploaded from Excel spreadsheets to the data tables previously described. There are constraints around the format of the spreadsheets for batch results for particular assays, which were designed around the existing format of reports being generated in our laboratory. Generally, samples are reported in rows, with analytes in columns. Metadata spreadsheets can be more flexible in format, allowing virtually any kind of clinical and demographic data to be displayed in columns, with persons or samples in rows.

The system makes no assumptions about the order in which batch results or experiment metadata are loaded. Once batch results and experiment metadata have been loaded into the database, connections between aliquot identifiers and sample identifiers and between sample identifiers and person identifiers can be established. Screen capture videos of representative workflows are included in the Additional file 1: Video S1, Additional file 2: Video S2, Additional file 3: Video S3.

The Upload Batch Results page allows the user to select a spreadsheet containing assay results and classify the file according to attributes such as operator, run date, assay, instrument, tissue, and time point. Upon upload, the spreadsheet is parsed and loaded into the database. Custom Java code parses the spreadsheets, handling lab-specific data organization for each assay. The original file is also posted on a web-based project management system (Basecamp, http://www.basecamphq.com) for future reference.

**Figure 2:**
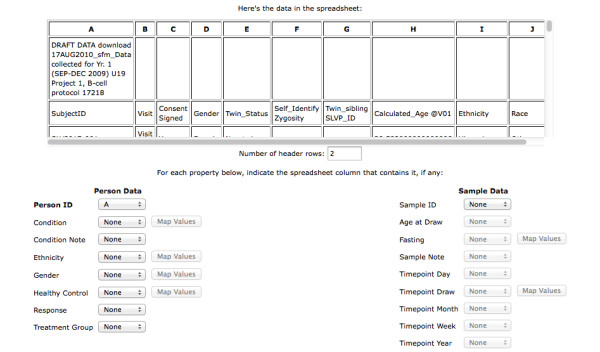
**Upload Experiment Metadata page**. A spreadsheet containing clinical and/or demographic "metadata" is uploaded and its contents displayed at the top of the page. In the lower half of the page, one can select which columns of the spreadsheet represent specific "Person" or "Sample" attributes. In this example, Person ID has been specified and the associated attributes are active. Sample ID has not been specified, so those attributes are inactive. Once an attribute has been mapped to a column, the associated Map Values button is activated (see Gender).

**Figure 3:**
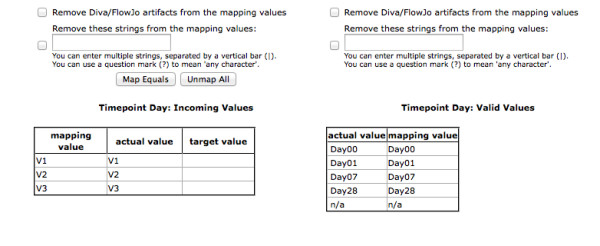
**Map Valid Values page**. The terms in certain columns (e.g. Condition, Ethnicity, and Gender) are mapped against a set of defined "valid values" for that attribute. In this way, a controlled vocabulary may be maintained even when terminology on incoming spreadsheets varies (e.g., "M" and "F" versus "male" and "female", "SLE" versus "lupus", etc.).

The Upload Experiment Metadata page, illustrated in Figure 2, allows the user to add clinical and/or demographic data from a spreadsheet. The only format constraint is that records be in rows with attributes in columns. The user maps spreadsheet columns to underlying database columns. Attributes associated with person (e.g. Condition, Ethnicity, and Gender) are displayed in the left hand column. Those associated with sample are displayed in the right hand column. Once the user specifies the spreadsheet column that contains either Person ID or Sample ID, the relevant attributes are activated for selection and mapping. Attributes with associated Map Values buttons are validated against a list of valid values. After selecting Map Values for a particular attribute, the user is presented with the Map Values page, as shown in Figure 3. This page supports drag and drop mapping of incoming values to a controlled vocabulary of valid values. Some valid values are specified at a global level (e.g. Condition, Ethnicity, and Gender), while others are project-specific (e.g. Response, Treatment, and Timepoint). Once the user has mapped values for all relevant attributes the data may be uploaded.

**Figure 4:**
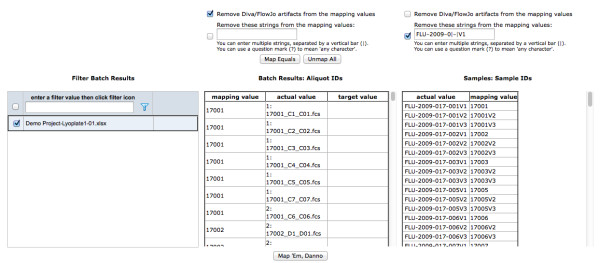
**Map Batch Results to Sample page**. Once sample metadata is uploaded, it needs to be "mapped" to laboratory data (batch results). This page allows the user to select a set of batch results (lower left), then map the aliquot IDs in those batch results to sample IDs from the uploaded metadata. Often, these don't fully match due to prefixes and suffixes that were added by the laboratory analysis software, so controls at the top of this page help to quickly remove such "artifacts". Once the IDs are made to match, the "Map Equals" button allows all matching IDs to be mapped at once. Alternately, individual IDs can be dragged and dropped from the Sample ID column to the Batch Results column to map them.

The Map Batch Results to Metadata (Figure 4) and the Map Sample to Person pages (not shown) support these identifier mapping tasks. The interface allows the user to drag values from the right hand column (e.g. Sample IDs) to the left hand column (e.g. Aliquot IDs). Values on the left hand side can be filtered by incoming document, limiting the mapping task to a certain report (e.g. the one just uploaded) or set of reports (e.g. Luminex reports). Furthermore, removal of character phrases from either list facilitates the derivation of equal values from both lists, allowing the users to take advantage of the Map Equals button. When the user confirms the mappings, the database IDs in the child table are updated with the database IDs for the mapped values in the parent table (e.g. aliquot_fact is updated with sample_id).

### Investigator-driven data exploration

The third component of a data warehouse is a set of end-user tools for data exploration and analysis. To support investigator-driven exploration, we deployed JasperServer, an open source business intelligence suite. We used this suite to provide an on-line analytical processing (OLAP) interface for our integrated data. OLAP provides a means of organizing data and an associated user interface that is designed to support interactive exploratory data analysis. Starting with a relatively simple table summarizing the data, the user can drill down into the data. The user can also drill through into the underlying detail. OLAP has been used in the biological sciences to support analysis of time series gene expression data [6], protein folding simulation results [7], and climate-related health vulnerabilities [8].

**Figure 5:**
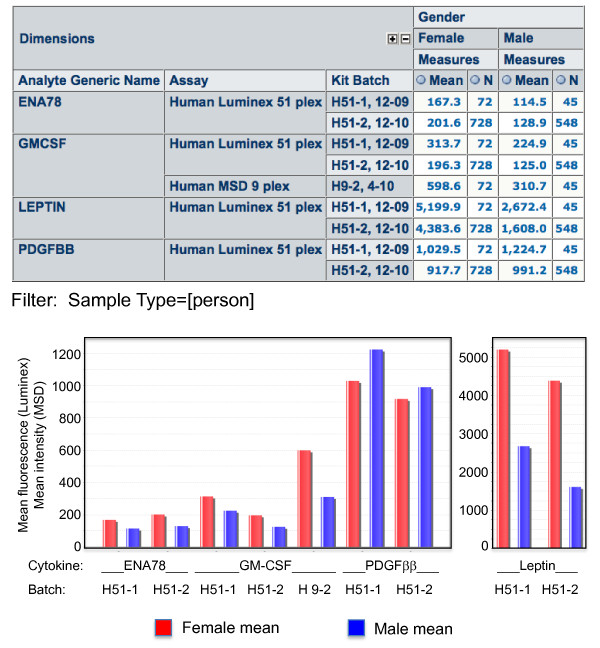
**(A) Assay group by Sample Day**. This OLAP cube displays aggregated data from each of three different assay types, and groups them by time point (day 0, 7, and 28). It is immediately apparent which assays were performed at which time points. (B) Identification of cytokines with gender bias. Expanding the Analytes dimension, and diplaying Gender in columns, we have selected those cytokines (4 of 51 analyzed) that show > 20% difference in mean expression between males and females. By also displaying Lot # in rows, we can see that the trends are preserved across two different lots of Luminex kits (H51-1 and H51-2), although absolute values vary by lot. For GM-CSF, the trend is also seen in a different type of assay (MSD 9-plex, shown as lot H9-2, 4/10). Other cytokines were not run in this assay type, so the cross-platform comparison can only be made for GM-CSF. Gender biases of the type shown here have been previously reported for ENA-78 [9], leptin [10,11], and PDGF [12], but to our knowledge not for GM-CSF. "Mean" indicates the mean MFI (median fluorescence intensity) of the indicated number of samples (N). Graphs of the data are exported from the native application to demonstrate the graphing functions of SDM.

The OLAP logical model organizes data in a multidimensional "cube," with facts (also known as measures) described by dimensions. Thus, our OLAP model is an extension of our dimensional data model as described above. One starting view of the data is shown in Figure 5A. This has been filtered on 2009 and 2010 data from our influenza vaccine study described in the Introduction. From this view, it can be readily seen which assays were done at which timepoints, and how many data points are available. The investigator can expand analytes associated with any or all assays on the rows. Notice that the time point dimension, which has been placed as a column, has already been expanded so that data for Day00, Day07, and Day28 are visible. A drill-down into specific Luminex analytes is shown in Figure 5B. Kit batch numbers have been added to the rows to further segregate the data by lot, and gender has been placed in the columns. "Mean" has been added as a Measure, in addition to "N" (number of data points). Graphs of the displayed data can be viewed and exported, as also shown at the bottom of Figure 5B. At these aggregate levels, the readout value is an average of all underlying data points. By clicking on the number in any particular cell, the investigator can see underlying data, as shown in Additional file 4: Table S1.

The results shown in Figure 5B serve to show how quickly a research question can be explored in SDM. In this case, we decided to explore possible gender differences in serum cytokine levels, using data from a large cohort of healthy participants. In a brief scan of aggregated mean immunoassay results by gender, 4 of 51 cytokines appeared to show a gender bias of > 20% (ENA-78, GM-CSF, leptin, and PDGFββ). Further segregating by lot number shows that the differences are reproducible across two lots of Luminex kits, and are also apparent, where tested, using a different type of assay (MesoScale Discovery). While not shown in the Figure, statistics like minimum, maximum, and median values can also be displayed to better judge the results. (We plan to add additional statistics, like SD and %CV in a future release.) Or, the calculated concentrations rather than raw values could be shown. And, with a few additional clicks, data can be segregated by age band to test whether there is an age bias as well. Given that there were no significant differences in these cytokines by age band, the gender effects shown do not appear to be confounded by an age effect, though we have not formally ruled this out. Interestingly, gender differences have been previously described for ENA-78 (CXCL5) [9], leptin [10,11], and PDGF [12], but not to our knowledge for GM-CSF. We are investigating whether the GM-CSF difference is reproduced in other studies, and what the basis of this difference might be.

**Figure 6:**
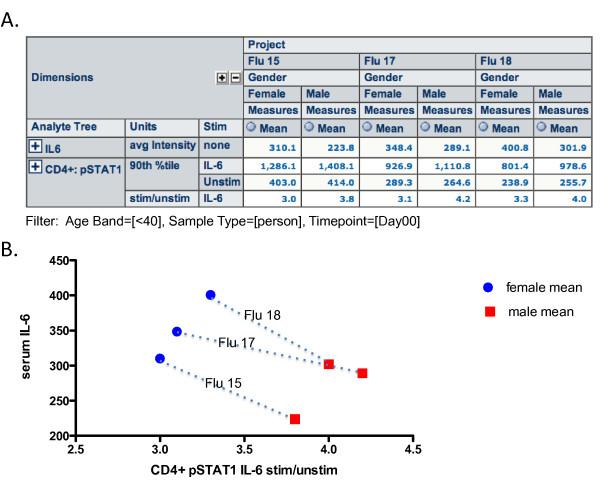
**OLAP integration of data from disparate types of assays and multiple projects, with demographic metadata**. (A) Serum IL-6 as measured by MSD assay is compared to CD4+ pSTAT1 expression as measured by phosphoepitope flow cytometry (baseline and IL-6-stimulated pSTAT1 MFI, as well as fold-change (stim/unstim) are all shown). Data for three healthy cohorts (Projects) are displayed in columns, broken out by gender. (B) Graphing the mean serum IL-6 levels as a function of mean CD4+ pSTAT1 IL-6 stim/unstim ratios for males and females in each study shows higher serum IL-6 means in females, and correspondingly lower pSTAT1 induction in response to IL-6 stimulation in CD4+ T cells. A possible hypothesis is that chronically high IL-6 levels in females result in poorer pSTAT1 induction in response to IL-6.

We have also used SDM to find relationships in data across assay platforms in our influenza study. For example, while we did not find a significant gender difference for serum IL-6 by Luminex, we did find such a difference using a more sensitive chemiluminescence assay (MSD). Furthermore, this difference, in which females had a higher mean IL-6 level than males, corresponded to a reduced mean induction of pSTAT1 in response to IL-6 in CD4+ T cells, in females versus males (Figure 6). As seen in the Figure, females had similar or slightly higher baseline pSTAT1 levels, and reduced induction with IL-6 stimulation (seen by reduced 90^th ^percentile fluorescence values and reduced stim/unstim ratios). These differences were consistent across three different cohorts (Projects), as segregated in the columns of Figure 6A. The results are shown graphically in Figure 6B. The obvious hypothesis, which remains to be tested, is that chronically high levels of IL-6 lead to reduced inducibility of pSTAT1 upon IL-6 stimulation. These data highlight the ability of SDM to aggregate data from dissimilar assays in the same view, across the same cohorts, to easily find relationships that may have biological relevance.

Of course, potential findings from SDM, such as those above, need to be followed up with additional tests, such as determination of statistical significance and correlation. Further studies, to test the hypotheses generated, may also be warranted. But SDM can be used as a starting point to find relationships and generate hypotheses that can eventually lead to new mechanistic understandings, and development of new diagnostics and therapeutics.

## Discussion

One of our goals was to allow investigators without advanced computational skills to interact more fully with their data, allowing them to explore large and heterogeneous data sets easily. We have been able to achieve this using an OLAP interface that enables investigators to formulate and test hypotheses in real time. As an example, one can display serum cytokine results in rows, and gender in columns; it then becomes a simple task to scan the rows to look for gender-specific cytokine differences. Upon finding such, a quick drill-through can determine whether these differences are the result of rare outliers or are pervasive. The built-in graphing functions of JasperServer can also aid in this sort of simple data mining.

Another goal of the project was to integrate data across projects. Thus, if the same standardized assays were applied in multiple studies, one can compare analytes in disease X to those in disease Y. Or, one can draw a set of age- and gender-matched controls from one study and compare them to patients with disease from another study.

The SDM interface was not designed to be an advanced statistical tool, nor a suite with sophisticated visualization functions. Instead, our goal here was to provide simple ways to filter and display data sets of interest, which can then be extracted for further analysis in tools such as R (a language and environment for statistical computing and graphics), Spotfire (data visualization software), or custom applications. Because the data is first integrated into the dimensional model in a relational database, data is accessible both through the OLAP interface and through these other tools. In addition, because of its use of the popular and pervasive programming language Java and open-source software products, the implementation facilitates future integration with other technologies of interest in this space. One example is Weka, a collection of machine learning algorithms for data mining tasks, such as decision trees [13]. Notably, these general-purpose data analysis tools are assay agnostic. In many cases, the same tools can be used to analyze data from multiple assays, and from the integrated data set. This reduces the need for investigators to familiarize themselves with platform-specific data analysis tools and algorithms.

One caveat of data aggregation is the need to account for assay differences and batch effects. For example, integration of data across sites requires standardization, or at least harmonization, of the assay protocols and readouts. Efforts to this effect are underway, for example, for flow cytometry immunophenotyping [14]. Even within a single lab, batch effects can be pronounced, particularly for functional assays such as phosphoepitope flow cytometry. Batch normalization routines may be required to accurately compare data from different experiments. We plan to integrate such routines into SDM in future work.

The use of both open-source components and cloud servers contributed to rapid deployment and low ongoing cost structure. Cloud servers can be procured in minutes. Open source software components (MySQL, JBoss, JasperServer) are readily available for installation and configuration. In addition, once software is installed and configured on one server, additional servers can be cloned from the disk image of the first server.

A limitation of this system is the fact that it currently deals only with data post-processing (e.g., gated flow cytometry output, or analyzed Luminex reports). In an ideal system, raw data files would be linked with processed data, such that re-processing could update the data in real time. Such systems exist for single-platform data (e.g., Cytobank for flow cytometry [15], and Stanford Microarray Database (SMD) for gene expression data [16]). However, these databases, while extremely useful, are focused on single-experiment analysis, rather than aggregation across many experiments; and they are compatible with only a single data platform. SDM could be expanded to provide links to raw data files, possibly through interfaces to CytoBank and SMD. The Data Miner is unlikely to itself become a tool for initial data analysis and feature extraction (e.g. gating of flow cytometry files or microarray image quantification), given the disparate data types and existing dedicated software for analysis of such raw data.

Similarly, SDM does not provide complete experimental protocols or details of sample processing, such as might be found in an electronic notebook. Instead, the batch run date provides a pointer to wherever such information is kept. Future development might involve linking SDM to an electronic notebook system, so that retrieval of this type of information becomes seamless.

## Conclusion

In conclusion, we have demonstrated that standard techniques of data warehousing can be applied to integrate and analyze heterogeneous immunological data from human participants. Within the first 4 months of operation, 2.8 million rows of data representing results from 237 sets of batch results across 46 projects were loaded into SDM. Furthermore, by leveraging open-source business intelligence software for investigator-driven data exploration and by deploying on cloud servers, we were able to meet our design goal of delivering a functional system within a reasonable time frame and cost (< 1 year and < $100,000; new instances based on the same code would obviously be much faster and cheaper). We also delivered a system that allows users to enter, map, and explore their data without relying on programmer intervention. We felt that this was critical to our philosophy of allowing biologists to interact with their data as much as possible. Finally, we achieved our design goal of accommodating multiple projects and investigators, which allows for data integration across assay platforms and studies. Thus, our system facilitates collaboration and re-use of data via comparison with new data sets over time. This is critical to the optimal use of large data sets consisting of disparate data types, which are becoming common in systems-level studies.

## Competing interests

JCS and WM are owners of CytoAnalytics.

## Authors' contributions

JCS and HTM conceived of and designed the software system. JCS and WM implemented the system. YRH designed and implemented the immunoassays, and performed data entry. MMD conceived of and designed the immune monitoring studies. JCS, HTM, and WM drafted the manuscript. All authors read and approved the final manuscript.

## Supplementary Material

Additional file 1 **Video S1 Screen capture video of Sherpa interface for uploading batch results**. Click here for file

Additional file 2 **Video S2, Screen capture video of Sherpa interface for uploading experiment metadata**. Click here for file

Additional file 3 **Video S3, Screen capture video of Sherpa interface for mapping batch results to samples**. Click here for file

Additional file 4 **Table S1**. Click here for file
